# Administration of fibrinogen concentrate combined with prothrombin complex maintains hemostasis in children undergoing congenital heart repair (a long‐term propensity score‐matched study)

**DOI:** 10.1111/aas.13945

**Published:** 2021-07-26

**Authors:** Corinna Velik‐Salchner, Helmuth Tauber, Verena Rastner, Werner Pajk, Markus Mittermayr, Dieter Wally, Juliane Kilo, David Vondrys, Dietmar Fries, Josef Fritz, Werner Streif

**Affiliations:** ^1^ Department of Anaesthesiology and Intensive Care Medicine Medical University of Innsbruck Innsbruck Austria; ^2^ Department of Cardiac Surgery Medical University of Innsbruck Innsbruck Austria; ^3^ Department of Medical Statistics Informatics and Health Economics Medical University of Innsbruck Innsbruck Austria; ^4^ Department of Paediatrics Medical University of Innsbruck Innsbruck Austria

**Keywords:** cardiopulmonary bypass management, coagulation factors, congenital heart disease, fibrinogen concentrate, prothrombin complex

## Abstract

**Background:**

Bleeding is a common problem in children with congenital heart disease undergoing major cardiac surgery requiring cardiopulmonary bypass (CPB). Little is known about optimal management with blood products.

**Objective:**

To investigate clinical outcome and hemostatic effects of fibrinogen concentrate (FC) in combination with prothrombin complex concentrate (PCC) versus standard treatment with fresh frozen plasma (FFP) in children undergoing cardiac surgery.

**Methods:**

For this single‐institution cohort study, data on 525 children were analyzed. Propensity score matching in 210 children was applied to reduce the impact of various baseline characteristics.

**Results:**

Three children treated with FC/PCC developed surgical site bleeding requiring surgical revision. One child developed central venous line‐related thrombosis. Blood loss through chest tube drainage was independent of FC/PCC. Coagulation abnormalities were not present in any of these children. Time to extubation and ICU stay did not differ. In the FC/PCC group, children received (median, Q1, Q3) 52 mg/kg (32, 83) FC and 28IU/kg (13, 44) PCC. Fibrinogen concentration was comparable at baseline. On admission to the ICU, fibrinogen was higher in children receiving FC/PCC, namely, 232 mg/dL (196, 280), than in children receiving FFP (186 mg/dL, 149, 224; *P* < .001). On discharge from the ICU, values did not differ ((FC/PCC 416 mg/dL (288, 501)), non‐FC/PCC 418 mg/dL (272, 585; *P* = 1.000)).

**Conclusion:**

FC/PCC was well tolerated and permitted hemostasis to be maintained, even in the very young. We were not able to detect a signal for inferiority of this treatment. We conclude that FC/PCC can safely replace FFP.

**Editorial Comment**

This single center retrospective study demonstrates that fibrinogen concentrate combined with prothrombin complex can be effectively used as an alternative to fresh frozen plasma in children undergoing congenital cardiac surgery.

## 1 INTRODUCTION

Bleeding is a common problem in children undergoing major cardiac surgery requiring cardiopulmonary bypass (CPB).^1^, ^2^, ^3^, ^4^ Selection and quantity of blood products used in these severely ill children differ significantly between centers.^5^, ^6^, ^7^ Information on optimal monitoring and management of hemostasis during congenital heart surgery in children is scarce in the literature. Encouraging outcomes in children and adults receiving fibrinogen led to integration of fibrinogen concentrates in the treatment algorithm at our institution.^6^, ^8^, ^9^, ^10^, ^11^, ^12^, ^13^ The aim of our study was to exploratively investigate the hemostatic effects of fibrinogen concentrate (FC) and prothrombin complex concentrate (PCC) treatment of children with congenital heart disease when undergoing heart surgery requiring CPB. Primary endpoint was the hemostatic effects of FC/PCC compared to treatment with FFP. Data collection and analyses included bleeding, thrombosis, chest tube drainage (CTD), need for blood products, time to extubation, duration of ICU stay, fibrinogen levels, and PT/aPTT at selected time points.

## 2 PATIENT SELECTION AND STUDY DESIGN

In May 2002, targeted administration of coagulation factor concentrates including fibrinogen concentrate (FC) and prothrombin complex concentrate (PCC) was introduced into clinical routine at our institution. For this single‐institution cohort study, all children undergoing congenital heart surgery requiring cardiopulmonary bypass (CPB) were included in this retrospective analysis. Medical records and laboratory reports for all children (0‐18 years) who required heart surgery on pump from December 2000 to December 2014 at the Medical University of Innsbruck were reviewed (n = 942). During the entire study period, two chief surgeons were responsible for planning and performing all procedures. A small team of anesthesiologists was responsible for performing congenital heart surgery using CPB and had developed in‐house guidelines and followed them rigorously.

**FIGURE 1:**
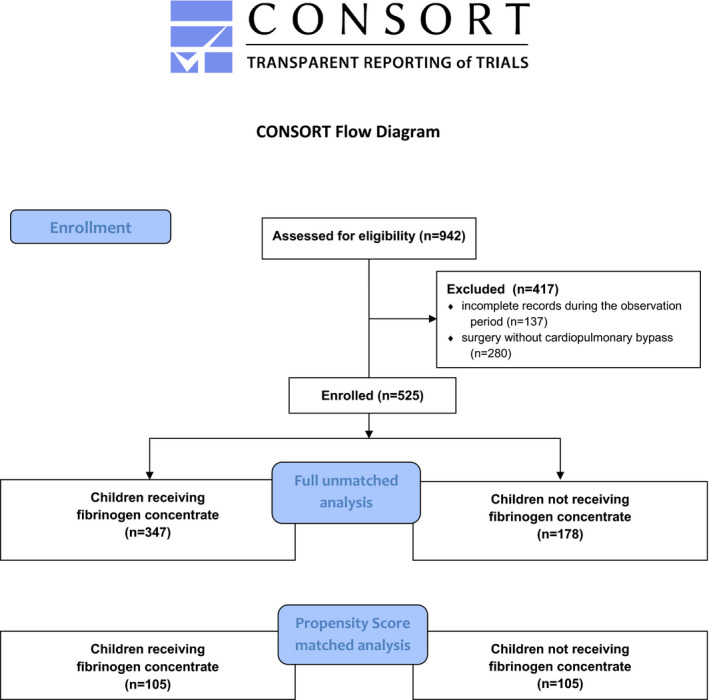
Study flowchart [Colour figure can be viewed at wileyonlinelibrary.com]

Children who did not need CPB (n = 280) and children with incomplete medical reports (n = 137) were excluded, leaving 525 children in our final analysis population. Of these 525 children, 347 (66.1%) received FC/PCC, while 178 (33.9%) did not receive FC/PCC (Fig. 1). The study was approved by the Ethics Committee of the Medical University of Innsbruck (AN2014‐0049 334/4.12, 356/5.2 (3708a); Chairperson Prof P. Lukas) on 24 March 2014.

## 3 ROUTINE MANAGEMENT

Anesthesia was induced with midazolam, ketamine, fentanyl and rocuronium and maintained with remifentanil and sevoflurane. For fluid substitution, Ringer's lactate and a modified 4% gelatin solution (Gelofusin^®^; Braun) were used. Children received 25 mg/kg methylprednisolone (up to 6 years, max 250 mg) and 30 mg/kg cefuroxime after induction of anesthesia. Routine monitoring included invasive arterial pressure measurement, electrocardiogram, pulse oximetry, capnography, near‐infrared spectroscopy (NIRS), and transesophageal echocardiography. All children had a central venous access.

Oxygenator size was chosen according to patient's weight and manufacturer's instructions (Supplementary Table oxygenator). Oxygenator and circuit were primed with Ringer's lactate 3 ml/kg, mannitol 15% 3 ml/kg, 20 ml/kg human albumin (Albiomin^®^ 50 g/l; Biotest Pharma GmbH), and 10 mg/kg tranexamic acid. For children weighing less than 15 kg, the priming volume was composed of washed and filtered packed red blood cells (PRBC) and fresh frozen plasma (FFP, 20 ml/kg). The target was hematocrit between 28% and 30% and normal colloid osmotic pressure. Children with more than 15 kg received clear priming. Potassium, sodium, calcium, and magnesium were added to balance electrolytes. Before starting CPB, mucosa heparin (Baxter AG) was administered at 300 U/kg to achieve an activated clotting time (ACT) above 480 s. Additional doses of 100 U/kg were administered to maintain ACT in the target range.

CPB was performed under mild hypothermia (35.4 ± 1.3℃). Pump flow rates were maintained between 2.4 L and 3.0 L/m^2^ to ensure a mean target perfusion pressure between 40 and 50 mmHg (in neonates between 30 and 50 mmHg). All children independent of age routinely underwent hemofiltration during CPB. We used multiBic^®^ hemofiltration solution 4 mmol/L potassium (Fresenius Medical Care) to replace intravascular volume. We aimed to control intravascular volume and prevent hemodilution and edema.

Myocardial protection was achieved with intermittent antegrade potassium colloid cardioplegia. Tranexamic acid was administered as follows: 10 mg/kg before CPB as loading dose and 10 mg/kg/h until wound closure. Children received protamine sulfate (Protamine; ICN Pharmaceuticals) after discontinuing CPB. PRBC were transfused if hematocrit was less than 28%‐30%. Apheresis platelet concentrate (APC, 20 ml/kg) was administered after weaning from CPB if platelet count was below 100 G/L or microvascular bleeding persisted. Fibrinogen concentrate (FC, Haemocomplettan^®^ P; CSL Behring GmbH) 50 mg/kg bw (repetitive dose 25 mg/kg) was administered to maintain fibrinogen levels >200 mg/dL. Prothrombin complex concentrate (PCC, Prothromplex Total 600 IU; Baxter) was combined with FC and repetitively administered if prolonged PT and aPTT (>1.5×) occurred. FFP was reserved for children with continuously prolonged PT and aPTT, even after PCC administration.

### 3.1 Demographic and clinical parameters

We recorded: age (months), body weight (bw, kg), gender, type of surgery, time on bypass (min), clamping time (min), time to extubation (hours), ICU stay (days), and chest tube drainage (CTD, ml/kg). CTD was measured every 24 h during ICU stay.

Any clinical complication of treatment was recorded.

### 3.2 Blood sampling

Blood samples were obtained exclusively from venous lines. Baseline blood samples were obtained the day before surgery (TP 1). Another blood sample was taken on arrival at the ICU (TP 2), after 24 h at the ICU (TP 3), and before discharge from the ICU, but latest after 8 days at the ICU (TP 4). Blood cell counts were measured from 1.2 ml tubes containing 1.6 mg ethylenediaminetetraacetic acid (EDTA)/ml blood (Sarstedt, Nuermbrecht, Germany). For coagulation tests, blood samples were collected in 1.8‐ml tubes containing 0.3 ml (0.106 mol/l) buffered (pH 5.5) sodium citrate (Sarstedt). Chemical analysis was performed from 1.2 ml tubes containing 35 U/ml lithium heparin.

### 3.3 Timing and Laboratory Analysis

Blood cell count was measured using the XE‐5000 Analyzer (Sysmex). Prothrombin time (Thromborel S; Siemens), partial thromboplastin time (PTT, Pathrombin SL; Siemens), fibrinogen (using the Clauss method, Multifibren U; Siemens) were measured with the BCS XP (Siemens). C‐reactive protein (CRP, CRPL3; Roche), creatinine (CREP2 assay) were measured with the COBAS 8000 (Roche).

### 3.4 Blood products

Administered blood products were counted: PRBC (ml/kg), FFP (ml/kg), apheresis platelet concentrate (APC, ml/kg), PCC IU/kg: Beriplex P/N 500 IU^®^ (CSL Behring); or Prothromplex Total 600 IU^®^ (Baxter), and FC (mg/kg, Haemocomplettan P 1g^®^; CSL Behring).

## 4 STATISTICAL ANALYSIS

Propensity score matching was used to reduce the impact of different baseline characteristics between children receiving FC/PCC and those receiving no such treatment. Relevant baseline variables, specifically sex, age, year of surgery (before 2003, 2004‐2007, 2008‐2011, 2012‐2014), weight, surgery type, time on bypass, clamping time, amount of FFP priming (ml/kg), and amount of PRBC priming (ml/kg), were entered into a logistic regression analysis with fibrinogen treatment as the dependent variable. FC was always combined with PCC, and therefore PCC was not included into the propensity score matching. From this model, a propensity score for each patient representing the probability of being treated with fibrinogen was derived as described by Austin.^14^ The resulting propensity score was used to create a 1:1 matched pair patient subsample, using the nearest‐neighbor matching method with a caliper width of 0.05. The balance of baseline variables between the study groups in the matched sample as well as differences in the outcome variables were assessed using the paired Wilcoxon test for continuous data, the McNemar test for dichotomous data, and the McNemar‐Bowker test for multinomial data. Differences in fibrinogen values over the course of time were evaluated using the Friedman test.

A significance level of α = 0.05 (two‐tailed) was applied for all P values. P values were corrected according to the Bonferroni method for parameters tested at multiple time points. Statistical analyses were performed using SPSS software, version 23 (IBM Corp.).

## 5 RESULTS

### 5.1 Patient characteristics, bypass data, outcome parameters, administered coagulation products, chest tube drainage, and surgery type in the Propensity Score‐matched patient population

Using propensity score matching, 105 children receiving FC/PCC were matched with another 105 children receiving no FC/PCC. In the FC/PCC group, children received 52 mg/kg (Q1: 32, Q3: 83) FC as compared to none in the other group (*P* < .001) (Table 1). Amount of PCC in the FC group was 28 IU/kg (12.5, 43.5) vs none in the other group (*P* < .001). All other parameters including chest tube drainage and surgery type did not significantly differ between groups. Time to extubation and duration of ICU stay did not differ. Three children in the FC/PCC group presented with clinically relevant bleeding requiring surgical revision. One overt thrombotic event occurred at the site of a left femoral vein catheter in a child receiving FC/PCC. Coagulation abnormalities were not present in any of these children. FC/PCC did not alter chest tube drainage.

**TABLE 1:** Body weight, bypass and outcome parameters, administered coagulation products, chest tube drainage, and surgery types in children receiving fibrinogen concentrate/PCC as compared to children not receiving fibrinogen concentrate/PCC in the Propensity Score‐matched patient population

	FC/PCC: yes (n = 105)	FC/PCC: no (n = 105)	*P* value*
Age (mon)	26 (7, 88)	38 (7, 120)	n.s.
Sex			n.s.
Male	55 (52.4%)	60 (57.1%)	
Female	50 (47.6%)	45 (42.9%)	
Body weight (kg)	10.7 (6.3, 21.9)	14.5 (6.3, 29.7)	n.s.
Bypass data			
Time on bypass (min)	111 (74, 165)	116 (76, 169)	n.s.
Clamping time (min)	42 (16, 79)	48 (19, 88)	n.s.
Outcome parameters			
Time to extubation (hours)	8.0 (4.0, 42.8)	7.0 (4.5, 23.0)	n.s.
ICU stay (days)	3 (2.0, 7.5)	3 (2.0, 5.5)	n.s.
Administered coagulation products			
FFP priming CPB (ml.kg^‐1^)	13.5 (0, 23.2)	6.0 (0, 21.3)	n.s.
FFP after CPB (ml.kg^‐1^)	0 (0, 0)	0 (0, 12.5)	n.s.
PRBC priming CPB (ml.kg^‐1^)	18.1 (0, 28)	11.5 (0, 28.9)	n.s.
PRBC after CPB( ml.kg^‐1^)	0 (0, 14.4)	0 (0, 7.9)	n.s.
Apheresis platelet concentrate after CPB (ml.kg^‐1^)	0 (0, 15)	0 (0, 18)	n.s.
FC after CPB (ml.kg‐1)	52 (32, 83)	0 (0, 0)	<.001
PCC after CPB(IU.kg^‐1^)	28 (12.5, 43.5)	0 (0, 0)	<.001
PRBC at the ICU (ml.kg^‐1^)	0 (0, 14.0)	0 (0, 13.5)	n.s.
CTD after 24 h (ml.kg^‐1^)	3.2 (1.7, 6.1)	3.9 (1.8, 6.7)	n.s.
Time to discharge from ICU (days)	3 (2.0, 7.5)	3 (2.0, 5.5)	n.s.
Surgery type			
Neonatal heart surgery	12 (11.4%)	10 (9.5%)	
Palliative surgery for univentricular heart defect	10 (9.5%)	4 (3.8%)	
Tetralogy of Fallot correction	4 (3.8%)	6 (5.7%)	
Complete/partial atrioventricular septal defect correction	6 (5.7%)	5 (4.8%)	n.s.
Atrial/ventricular septal defect closure	43 (41.0%)	41 (39.0%)	
Valve surgery	24 (22.9%)	33 (31.4%)	
Other	6 (5.7%)	6 (5.7%)	

Note

Data are shown as median (Q1, Q3) or as number (percentage); FC, fibrinogen concentrate; PCC, prothrombin complex concentrate; FFP, fresh frozen plasma; ICU, intensive care unit; FFP, fresh frozen plasma; PRBC, packed red blood cells; CTD, chest tube drainage.

* *P* value from Fisher's exact test for sex and surgery type, all other p values from Mann‐Whitney U tests.

### 5.2 Blood cell count, coagulation parameters, and C‐reactive protein in the Propensity Score‐matched patient population

Blood cell count, coagulation parameters, and CRP measured at baseline (T1), on arrival at the ICU (T2), after 24 h at the ICU (T3), and immediately before discharge from the ICU (T4) are shown (Table 2). At baseline, hemoglobin was comparable between groups. On admission to the ICU, after 24 h at the ICU and at discharge from the ICU, hemoglobin did not differ between groups. White blood cell count and platelet count did not differ between groups at any time point.

**TABLE 2:** Blood cell count, coagulation parameters, and C‐reactive protein compared between children receiving FC/PCC and children not receiving FC/PCC at baseline (T1), on arrival at the ICU (T2), after 24 hours at the ICU (T3) and immediately before discharge from the ICU (T4) in the Propensity Score‐matched patient population

Normal range	Hb 120‐157g.l^‐1^)	White blood cells 4.0‐10.0G.l^‐1^	PLT 150‐380G.l^‐1^	Fibrinogen 210‐400mg.dl^‐1^	PT 70%‐130%	PTT 26‐37s	CRP 0.0‐0.50mg.dl^‐1^
T1							
FC+PCC: yes (N = 105)	136 (124, 148)	8.1 (6.7, 10.8)	301 (246, 361)	255 (223, 301)	93 (84, 99)	35 (32, 37)	0.7 (0.7, 0.7)
FC+PCC: no (N = 105)	133 (121, 143)	8.0 (6.1, 9.4)	289 (232, 369)	253 (226, 301)	92 (83, 100)	35 (32, 39)	0.7 (0.7, 0.7)
	n.s.	n.s.	n.s.	n.s.	n.s.	n.s.	n.s.
T2							
FC+PCC: yes (N = 105)	125 (109, 137)	9.6 (8.1, 12.6)	149 (119, 190)	232 (196, 280)	76 (66, 86)	41 (36, 47)	0.7 (0.7, 0.7)
FC+PCC: no (N = 105)	116 (104, 127)	10.3 (7.2, 12.7)	147 (113, 179)	186 (149, 224)	64 (54, 72)	41 (37, 48)	0.7 (0.7, 0.7)
	n.s.	n.s.	n.s.	***P* < .001**	***P* < .001**	n.s.	n.s.
T3							
FC+PCC: yes (N = 105)	123 (108, 136)	12.0 (9.8, 14.0)	169 (133, 216)	307 (253, 350)	79 (70, 89)	38 (34, 44)	3.3 (2.1, 5.8)
FC+PCC: no (N = 105)	120 (106, 132)	11.3 (9.3, 12.9)	163 (132, 203)	279 (235, 318)	76 (66, 88)	38 (35, 42)	4.2 (2.5, 5.8)
	n.s.	n.s.	n.s.	***P* =.048**	n.s.	n.s.	n.s.
T4							
FC+PCC: yes (N = 105)	124 (109, 135)	10.0 (7.4, 12.8)	174 (121, 240)	416 (288, 501)	84 (74, 90)	38 (35, 47)	2.8 (1.3, 6.0)
FC+PCC: no (N = 105)	123 (104, 133)	10.0 (7.1, 12.0)	171 (133, 208)	418 (272, 585)	86 (63, 100)	38 (34, 44)	3.0 (1.4, 6.8)
	n.s.	n.s.	n.s.	n.s.	n.s.	n.s.	n.s.

Note

Data are presented as median (Q1, Q3).

Abbreviations: CRP, C‐reactive protein; FC, fibrinogen concentrate; Hb, hemoglobin; PCC, prothrombin complex concentrate; PLT, platelets; PT, prothrombin time; PTT, partial prothrombin time.

*P* values from paired Wilcoxon signed rank tests, corrected for multiple testing by multiplying the original p value by four, the number of time points, according to the Bonferroni method.

Bold to highlight statistically relevant *p*‐values.

**FIGURE 2:**
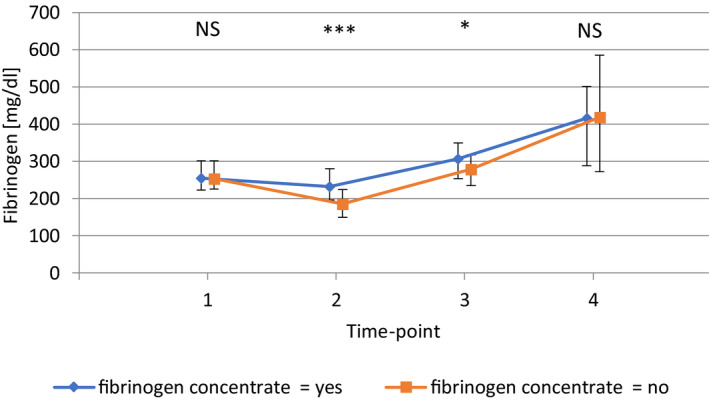
Fibrinogen course at baseline (T1), on arrival at the ICU (T2), after 24 hours at the ICU (T3) and immediately before discharge from the ICU (T4) in the Propensity Score–matched patient population. *P < .05; **P < .01; ***P < .001, n.s., not significant (P values from paired Wilcoxon signed‐rank tests, corrected for multiple testing by multiplying the original p value by four, the number of time points, according to the Bonferroni method); points represent the median, bars the interquartile range [Colour figure can be viewed at wileyonlinelibrary.com]

Fibrinogen concentration was comparable between groups at baseline. On admission to the ICU, fibrinogen concentration was higher in children receiving FC 232 mg/dL (196, 280) than in children without FC, namely, 186 mg/dL (149, 224; *P* < .001). After 24 h at the ICU, children receiving FC had higher fibrinogen levels, namely, 307 mg/dL (253, 350; *P* < .001), than did children without FC, who showed 279 mg/dL (235, 318; *P* = .048). On discharge from the ICU, values did not differ (*P* = 1.000), (Fig. 2). Regarding fibrinogen time course, the drop from baseline to admission to the ICU was significant in children without FC (*P* = .008), while it was not significant in the FC group (*P* = .628). After 24 h at the ICU, fibrinogen levels were again comparable to baseline values (p_w/o FC_ = 1.000, p_with FC_ = .075). Finally, at discharge from the ICU, fibrinogen levels were statistically significantly higher than baseline in both groups (p_w/o FC_ = .014, p_with_ _FC_ < .001).

Baseline PT values (%) were comparable between matched groups. After admission to the ICU, PT values were higher in children who had received FC/PCC (76% (66, 86; *P* < .001)) than in children without FC/PCC (64% (54, 72; *P* < .001)). PTT values and measured CRP did not differ between groups at any measurement points.

### 5.3 Patient characteristics, bypass data, outcome parameters, administered coagulation products, chest tube drainage, and surgery type in the full unmatched population

Two hundred and four children (m = 111; f = 93; neonates n = 65) were younger than 1 y, 187 children (m = 93; f = 94) were between 1 and 6 y and 134 children (m = 87; f = 47) were between 7 and 18 y . A total of 347 (66%) children received FC and 178 (34%) did not receive FC (Table S1). Children receiving FC were younger (*P* < .001), had a lower body weight (*P* < .001), longer time on cardiopulmonary bypass (*P* < .001), longer time to extubation (*P* < .001), and longer stay at the intensive care unit (ICU, *P* < .001). Amount of administered FC was 61 mg/kg (42, 98) vs none in the other group (*P* < .001). PCC was given in the FC group at 30 IU/kg (18, 47) vs none in the other group (*P* < .001). Platelet concentrate requirements did not differ between groups. Chest tube drainage was similar between groups. Relative frequency of different surgery types significantly differed between children receiving FC and children not receiving FC (*P* = .001). Specifically, neonatal heart surgery was more common in children receiving FC (*P* = .002), while atrial/ventricular septal defect closure was more common in children not receiving FC (*P* = .001).

### 5.4 Blood cell count, coagulation parameters, and C‐reactive protein in the full unmatched population

Blood cell count, coagulation parameters, and CRP measured at baseline (T1), on arrival at the ICU (T2), after 24 hours at the ICU (T3), and immediately before discharge from the ICU (T4) are shown in the full unmatched population (Table S2). At baseline, hemoglobin was comparable between groups. On admission to the ICU, Hb was higher in the FC/PCC group (*P* < .001). After 24 h at the ICU, Hb was still higher in the FC/PCC group (*P* = .008). On discharge from the ICU, hemoglobin did not differ between groups. Although clinically not relevant, white blood cell count was higher in children receiving FC/PCC at baseline (*P* < .001). At all other time points, white blood cell count did not differ between groups. Platelet count was comparable between groups at baseline, after admission to the ICU, after 24 h at the ICU, and at discharge from the ICU.

Fibrinogen concentration was comparable between groups at baseline. On admission to the ICU, fibrinogen concentration was higher in children receiving FC/PCC (*P* < .001). After 24 h at the ICU, children receiving FC/PCC still had higher fibrinogen levels (*P* = .004). On discharge from the ICU, fibrinogen was significantly lower in children who had received FC/PCC, namely, 349 mg/dl vs 442 mg/dl (*P* = .012) in children without FC/PCC (Fig. S1). PT values were comparable between groups at baseline. After admission to the ICU, PT in % values was higher in children who had received FC/PCC (*P* < .001). After 24 h at the ICU and at discharge, PT values were comparable between groups. Although not clinically relevant, PTT values were longer in children receiving FC/PCC at baseline (*P* = .012). On admission to the ICU, after 24 h and at discharge from the ICU values did not differ.

CRP levels did not differ between age groups at baseline or on admission to the ICU.

After 24 h at the ICU and at discharge, CRP levels were higher in children receiving no FC/PCC.

## 6 DISCUSSION

Bleeding is a common problem in children undergoing major cardiac surgery requiring cardiopulmonary bypass (CPB). Lack of commonly accepted guidelines results in administration of a wide variety of blood products in these children.^15^ Enrolled in our study were 525 children in order to compare treatment with FC/PCC to standard therapy with FFP; 210 children were analyzed by applying Propensity Score Matching. Three children treated with FC/PCC developed surgical site bleeding requiring surgical revision. One child developed central venous line‐related thrombosis. Blood loss through chest tube drainage was independent of administration of FC/PCC. Coagulation abnormalities were not present in any of these children. Fibrinogen levels were higher in children receiving FC until 24 h after surgery, but were comparable at discharge from the ICU. After CPB, administration of FC/PCC allowed us to replace FFP for correction of hemostasis. FC/PCC was well tolerated in this critically ill group of children undergoing major cardiac surgery requiring CPB.

Relevant bleeding following cardiac surgery usually occurs within 12‐24 h after admission to the ICU.^16^, ^17^ In our study, three children developed clinically relevant bleeding needing surgical revision. None of them had fibrinogen levels below the critical threshold of 150 mg/L at the occurrence of bleeding. Increased chest tube drainage is an indicator for a deranged hemostatic system following cardiac surgery. Although not significant, children receiving FC/PCC had a lower CTD.

The main concern regarding the use of FC/PCC is the possibility of thrombotic complications. In our study, only one central line‐related thrombotic event occurred, but was not attributable to the use of FC/PCC. Thrombotic events in children are multifactorial,^18^ but central lines are the most important cause of thrombosis in children.^19^ Of note, children in our cohort were not screened for thrombosis and we may have not captured all thrombotic events when symptoms were not overt. However, follow‐up investigations at the same institution did not reveal any further thrombosis.

In our large cohort of children, we investigated effects of FC/PCC represented by fibrinogen levels and PT/aPTT to maintain and restore hemostasis. FC/PCC provide an alternative to FFP for replacement of coagulation factors without unnecessary fluid overload; this is critical in small children because 15‐30 mL/kg of FFP is necessary to increase factor levels by more than 20%. At least theoretically FC/PCC is advantageous as compared to FFP. In general, there is a tendency to use more factor concentrates in this group of critically ill children.^16^, ^17^, ^20^, ^21^ In our study, administration of FC/PCC permitted fibrinogen to be maintained at constant and sufficient levels independent of age. Edema, fluid overload of the lung, and need for diuretics are typical sign of fluid overload; these findings were perceived independent of treatment group. Although not clinically relevant, a tendency to an increased need for PRBC was observed in the FC/PCC group. Children receiving exclusively FFP did not need more platelet transfusions. We detected no difference in clinical outcome in children receiving FC/PCC or standard therapy with FFP. We conclude that FC/PCC was well tolerated and permitted hemostasis to be restored and maintained and may be a safe alternative to FFP.^2^, ^5^, ^16^, ^17^, ^20^, ^22^, ^23^, ^24^

Guidelines issued by the European Society of Anesthesiology (ESA) recommend keeping the level of plasma fibrinogen at no <150‐200 mg/dL in bleeding patients.^25^ In our study, plasma fibrinogen levels in the FC/PCC group were kept within normal range to maintain hemostasis. However, outlayers of fibrinogen levels may not have been recorded. So far, further increased levels of fibrinogen have not been shown to have a greater protective effect on bleeding and blood loss.^23^ From this standpoint it is understandable that we recognized only non‐significant effects of higher fibrinogen levels on blood loss and bleeding.

In our study, fibrinogen levels were higher in children receiving FC/PCC until 24 h after surgery, but they were comparable between groups at discharge from the ICU. Authors suspect that targeted blood management with FC/PCC had an effect on endogenous fibrinogen release. This means that short‐time recovery of endogenous fibrinogen may be expected within 24 h in the absence of active bleeding after surgery independent of administered FC. This is in accordance with Erdoes,^26^ who investigated 26 adult patients in an observational study demonstrating 0.08 g/L per hour recovery of endogenous fibrinogen. In our cohort, children reached baseline fibrinogen levels within the first post‐operative day, independent of treatment with FC/PCC.

The hemostatic system of the very young differs in many aspects from that of older children. Coagulation factors achieve almost adult values at 6 months of age.^27^ Due to the relatively high volume in the external circuit during CPB, children <15 kg received weight‐adapted PRBC and FFP. Although different in various aspects, propensity score matching did not show clinically relevant differences in outcome (Table 1). We suspect that priming, severity of the underlying disease, underweight, and other peculiarities of these severely diseased children overruled age‐specific aspects of the coagulation system.

### 6.1 Limitations

The study design (cohort study) and the prolonged time interval of recruitment may have influenced results. However, all children were treated following strict in‐house guidelines; any changes were protocolled and critically appraised by authors. Although underpowered for conclusions to be drawn on the safety and effectiveness of FC/PCC, this cohort of 525 children reflects clinical experience over a long period.

All children that received FC also received PCC. The group of children treated with FC/PCC was younger and differed in many aspects. Propensity score matching was applied to accommodate this fact.

Another important potential confounder was priming of cardiopulmonary bypass systems with PRBC and FFP in younger children weighing <15 kg.^28^ Again, propensity score matching was applied to reduce the risk of misinterpreting findings.

## 7 CONCLUSION

We compared FC/PCC and FFP in a large cohort of critically ill children undergoing major cardiac surgery requiring cardiopulmonary bypass. FC/PCC was well tolerated and permitted hemostasis to be maintained, even in the very young. Over the years FC/PCC treatment has been established at our institution with no perceived and recorded increase in adverse events including the need for blood products and reoperation. We conclude that FC/PCC allows hemostasis to be restored and maintained.

## CONFLICT OF INTERESTS

C.V‐S. has received personal fees outside the submitted work from Boehringer Ingelheim (Vienna, Austria). MM and WS have received personal fees from CSL Behring GmbH outside the submitted work. DF has received grants and personal fees from CSL Behring, LFB, and Braun outside the submitted work.

## Supporting information

Supplementary Material

Supplementary Table Oxygenator

## References

[1] Faraoni D . Definition of postoperative bleeding in children undergoing cardiac surgery with cardiopulmonary bypass: One size doesn't fit all. J Thorac Cardiovasc Surg. 2018;155(5):2125‐2126. 29452707 10.1016/j.jtcvs.2018.01.017

[2] Faraoni D , Van der Linden P . Factors affecting postoperative blood loss in children undergoing cardiac surgery. J Cardiothorac Surg. 2014;9:32. 24512988 10.1186/1749-8090-9-32 PMC3924411

[3] Miller BE , Mochizuki T , Levy JH , et al. Predicting and treating coagulopathies after cardiopulmonary bypass in children. Anesth Analg. 1997;85(6):1196‐1202. 9390579 10.1097/00000539-199712000-00003

[4] Spiezia L , Di Gregorio G , Campello E , et al. Predictors of postoperative bleeding in children undergoing cardiopulmonary bypass: a preliminary Italian study. Thromb Res. 2017;153:85‐89. 28359027 10.1016/j.thromres.2017.03.021

[5] Faraoni D , Willems A , Romlin BS , et al. Development of a specific algorithm to guide haemostatic therapy in children undergoing cardiac surgery: a single‐centre retrospective study. Eur J Anaesthesiol. 2015;32(5):320‐329. 25387300 10.1097/EJA.0000000000000179

[6] Galas FRBG , de Almeida JP , Fukushima JT , et al. Hemostatic effects of fibrinogen concentrate compared with cryoprecipitate in children after cardiac surgery: a randomized pilot trial. J Thorac Cardiovasc Surg. 2014;148(4):1647‐1655. 24951020 10.1016/j.jtcvs.2014.04.029

[7] Lee JW , Yoo Y‐C , Park HK , et al. Fresh frozen plasma in pump priming for congenital heart surgery: evaluation of effects on postoperative coagulation profiles using a fibrinogen assay and rotational thromboelastometry. Yonsei Med J. 2013;54(3):752‐762. 23549826 10.3349/ymj.2013.54.3.752 PMC3635629

[8] Haas T , Fries D , Velik‐Salchner C , et al. Fibrinogen in craniosynostosis surgery. Anesth Analg. 2008;106(3):725‐731. 18292409 10.1213/ane.0b013e318163fb26

[9] Innerhofer P . Fibrinogen and prothrombin complex concentrate for the management of massive bleeding. Wien Klin Wochenschr. 2010;122(Suppl 5):S16‐17. 21598441

[10] Innerhofer P , Fries D , Oswald E , et al. Early fibrinogen‐concentrate administration in management of trauma‐induced coagulopathy – Authors' reply. Lancet Haematol. 2017;4(8):e348‐e349. 10.1016/S2352-3026(17)30126-6 28754192

[11] Innerhofer P , Westermann I , Tauber H , et al. The exclusive use of coagulation factor concentrates enables reversal of coagulopathy and decreases transfusion rates in patients with major blunt trauma. Injury. 2013;44(2):209‐216. 23000050 10.1016/j.injury.2012.08.047

[12] Miceli A , Ranucci M , Glauber M . Fibrinogen concentrate as first‐line hemostatic treatment for the management of bleeding in complex cardiac surgery. J Thorac Cardiovasc Surg. 2016;151(2):383‐384. 26470911 10.1016/j.jtcvs.2015.09.023

[13] Tauber H , Innerhofer P , Breitkopf R , et al. Prevalence and impact of abnormal ROTEM(R) assays in severe blunt trauma: results of the 'Diagnosis and Treatment of Trauma‐Induced Coagulopathy (DIA‐TRE‐TIC) study'. Br J Anaesth. 2011;107(3):378‐387. 21705350 10.1093/bja/aer158

[14] Austin PC . An introduction to propensity score methods for reducing the effects of confounding in observational studies. Multivariate Behav Res. 2011;46(3):399‐424. 21818162 10.1080/00273171.2011.568786 PMC3144483

[15] Faraoni D . Fibrinogen concentrate as first‐line therapy in children undergoing cardiac surgery: promising perspectives. J Thorac Cardiovasc Surg. 2015;149(5):1466‐1467. 25983258 10.1016/j.jtcvs.2014.07.022

[16] Dennhardt N , Sümpelmann R , Horke A , et al. Prevention of postoperative bleeding after complex pediatric cardiac surgery by early administration of fibrinogen, prothrombin complex and platelets: a prospective observational study. BMC Anesthesiol. 2020;20(1):302. 33339495 10.1186/s12871-020-01217-1 PMC7747387

[17] Ranucci M , Bianchi P , Cotza M , et al. Fibrinogen levels and postoperative chest drain blood loss in low‐weight (<10 kg) children undergoing cardiac surgery. Perfusion. 2019;34(8):629‐636. 31250738 10.1177/0267659119854246

[18] Sherrod BA , McClugage SG , Mortellaro VE , et al. Venous thromboembolism following inpatient pediatric surgery: analysis of 153,220 patients. J Pediatr Surg. 2019;54(4):631‐639. 30361075 10.1016/j.jpedsurg.2018.09.017 PMC6451662

[19] van Ommen CH , Sol JJ . Developmental hemostasis and management of central venous catheter thrombosis in neonates. Semin Thromb Hemost. 2016;42(7):752‐759. 27637010 10.1055/s-0036-1592299

[20] Machovec KA , Jooste EH . Pediatric transfusion algorithms: coming to a cardiac operating room near you. J Cardiothorac Vasc Anesth. 2019;33(7):2017‐2029. 30686658 10.1053/j.jvca.2018.12.008

[21] Rizza A , Romagnoli S , Ricci Z . Fluid status assessment and management during the perioperative phase in pediatric cardiac surgery patients. J Cardiothorac Vasc Anesth. 2016;30(4):1085‐1093. 26944520 10.1053/j.jvca.2015.11.007

[22] Faraoni D , Willems A , Melot C , et al. Efficacy of tranexamic acid in paediatric cardiac surgery: a systematic review and meta‐analysis. Eur J Cardiothorac Surg. 2012;42(5):781‐786. 22531271 10.1093/ejcts/ezs127

[23] Faraoni D , Willems A , Savan V , et al. Plasma fibrinogen concentration is correlated with postoperative blood loss in children undergoing cardiac surgery. A retrospective review. Eur J Anaesthesiol. 2014;31(6):317‐326. 24503704 10.1097/EJA.0000000000000043

[24] Faraoni D , Zurakowski D , Vo D , et al. Post‐operative outcomes in children with and without congenital heart disease undergoing noncardiac surgery. J Am Coll Cardiol. 2016;67(7):793‐801. 26892415 10.1016/j.jacc.2015.11.057

[25] Kozek‐Langenecker SA , Ahmed AB , Afshari A , et al. Management of severe perioperative bleeding: guidelines from the European Society of Anaesthesiology: First update 2016. Eur J Anaesthesiol. 2017;34(6):332‐395. 28459785 10.1097/EJA.0000000000000630

[26] Erdoes G , Dietrich W , Stucki MP , et al. Short‐term recovery pattern of plasma fibrinogen after cardiac surgery: a prospective observational study. PLoS One. 2018;13(8):e0201647. 30075017 10.1371/journal.pone.0201647 PMC6075772

[27] Kurnik K , Bidlingmaier C , Hutker S , et al. Haemostatic disorders in children. Hamostaseologie. 2016;36(2):109‐125. 26988657 10.5482/HAMO-15-04-0016

[28] Bianchi P , Cotza M , Beccaris C , et al. Early or late fresh frozen plasma administration in newborns and small infants undergoing cardiac surgery: the APPEAR randomized trial. Br J Anaesth. 2017;118(5):788‐796. 28510741 10.1093/bja/aex069

