# Supplementary material for: Administration of fibrinogen concentrate combined with prothrombin complex maintains hemostasis in children undergoing congenital heart repair (a long‐term propensity score‐matched study)
Source: Acta Anaesthesiol Scand. 2021 Jul 26;65(9):1178–86. doi: 10.1111/aas.13945 (PMC8518112; doi:10.1111/aas.13945)
Supplement: Supplementary file 1 — Supplementary Material [file AAS-65-1178-s002.docx]

**Supplementary Table 1:** Age, body weight, bypass and outcome parameters, administered coagulation products, chest tube drainage and surgery type for children receiving FC/PCC as compared to children not receiving FC/PCC in the full unmatched population.

|  | **FC/PCC: yes FC/PCC: no** | |  |
| --- | --- | --- | --- |
|  | **(n = 347)** | **(n = 178)** | **p value*** |
| **Age (mon)**  **Sex**  male  female  **Body weight (kg)** | 14 (4, 54)  188 (54.2%)  159 (45.8%)  8.3 (5.1, 17) | 54 (14, 133)  103 (57.9%)  75 (42.1%)  16.7 (8.8, 36.1) | <0.001  n.s.  <0.001 |
| **Bypass data** |  |  |  |
| Time on bypass (min) | 145 (96, 206) | 100 (68, 154) | <0.001 |
| Clamping time (min) | 53 (8, 95) | 38 (20, 81) | n.s. |
|  |  |  |  |
| **Outcome parameters** |  |  |  |
| Time to extubation (hours) | 12 (5, 109) | 8 (5, 18) | <0.001 |
| ICU stay (days) | 6 (2, 12) | 2 (2, 5) | <0.001 |
|  |  |  |  |
| **Administered coagulation products** |  |  |  |
| FFP priming CPB (ml.kg^-1^) | 16 (0, 26) | 0 (0, 15) | <0.001 |
| FFP after CPB (ml.kg^-1^) | 0 (0, 0) | 0 (0, 8) | n.s. |
|  |  |  |  |
| PRBC priming CPB (ml.kg^-1^) | 21 (0, 32) | (0, 23) | <0.001 |
| PRBC after CPB (ml.kg^-1^) | 0 (0, 15) | 0 (0, 7) | <0.001 |
|  |  |  |  |
| Apheresis platelet concentrate after CPB (ml.kg^-1^) | 0 (0, 16) | 0 (0, 18) | n.s. |
|  |  |  |  |
| Fibrinogen concentrate after CPB (ml.kg-1) | 61 (42, 98) | - | <0.001 |
| PCC after CPB (IU.kg^-1^) | 30 (18, 47) | - | <0.001 |
| **CTD after 24h** (**ml.kg^-1^)** | 4.3 (1.9, 6.9) | 4.7 (2.1, 7.1) | n.s. |
| **Surgery Type** |  |  |  |
| Neonatal heart surgery | 54 (15.6%) | 11 (6.2%) |  |
| Palliative surgery for univentricular heart defect | 24 (6.9%) | 6 (3.4%) |  |
| Tetralogy of Fallot correction | 24 (6.9%) | 10 (5.6%) |  |
| Complete/partial atrioventricular septal defect correction | 23 (6.6%) | 8 (4.5%) | 0.001 |
| Atrial/ventricular septal defect closure | 100 (28.8%) | 77 (43.3%) |  |
| Valve surgery | 85 (24.5%) | 56 (31.5%) |  |
| Heart transplantation/ventricular assist device | 14 (4.0%) | 2 (1.1%) |  |
| Other | 23 (6.6%) | 8 (4.5%) |  |

*p value from Fisher’s exact test for sex and surgery type, all other p values from Mann-Whitney U tests.

Data are shown as median (Q1, Q3) or as number (percentage); FC, fibrinogen concentrate; PCC, prothrombin complex concentrate; ICU, intensive care unit; FFP, fresh frozen plasma; PRBC, packed red blood cells; CTD, chest tube drainage.

**Supplementary Table 2:** Blood cell count, coagulation parameters and C-reactive protein at baseline (T1), on arrival at the ICU (T2), after 24 hours at the ICU (T 3) and immediately before discharge from the ICU (T4) in the full unmatched population.

| **normal range** | **Hb**  **120–157g.l^-1^)** | **White blood**  **cells**  **4.0-10.0G.l^-1^** | **PLT**  **150-380G.l^-1^** | **Fibrinogen**  **210-400mg.dl^-1^** | **PT**  **70-130%** | **PTT**  **26-37s** | **CRP**  **0.0-0.50mg.dl^-1^** |
| --- | --- | --- | --- | --- | --- | --- | --- |
| **T1  FC+PCC:yes(N=347) FC+PCC: no (N=178)** | 132(120,145)  131(121,142)  *n.s.* | 8.9(7.0, 11.4)  7.8(6.0, 09.5)  ***p<0.001*** | 288(231,354)  299(245,371)  *n.s* | 245(216,297)  253(228,300)  *n.s.* | 89(78,98)  92(83,98)  *n.s.* | 36(33,41)  35(33,38)  ***p=0.012*** | 0.7(0.7, 0.7)  0.7(0.7, 0.7)  *n.s* |
| **T2  FC+PCC: yes(N=347) FC+PCC: no (N=178)** | 125(112,136)  111(101,123) ***p<0.001*** | 9.4(7.1, 12.5)  10.2(7.6, 12.4)  *n.s.* | 149(119,200)  152(117,182)  *n.s* | 233(208,269)  171(134,215) ***p<0.001*** | 73(62,83)  62(53,70)  ***p<0.001*** | 40(36,48)  41(37,48)  *n.s.* | 0.7(0.7, 0.7)  0.7(0.7, 0.7)  *n.s* |
| **T3  FC+PCC: yes(N=347) FC+PCC: no (N=178)** | 123(108,138)  116(105,128)  ***p=0.008*** | 11.8(9.4, 14.2)  11.3(9.3, 13.1)  *n.s.* | 167(130,213)  170(136,205)  *n.s* | 290(246,346)  265(236,316)  ***p=0.004*** | 74(66,85)  74(65,85) *n.s.* | 39(35,45)  38(43,41) *n.s.* | 3.3(2.0, 5.5)  4.4(2.6, 6.4)  ***p=0.028*** |
| **T4 FC+PCC: yes(N=347) FC+PCC: no (N=178)** | 122(109,135)  116(104,131)  *n.s.* | 9.2(7.2, 12.3)  10.0(7.4, 12.2)  *n.s.* | 192(141,253)  178(136,219) *p=n.s.* | 349(228,483)  442(285,585) ***p=0.012*** | 83(70,93)  86(69,99)  *n.s****.*** | 39(35,49)  36(33,44) *n.s.* | 2.0(1.0, 4.4)  3.4(1.8, 9.5)  ***p<0.001*** |

Data are presented as median (Q1, Q3)

FC, fibrinogen concentrate; PCC, prothrombin complex concentrate; Hb, hemoglobin; PLT, platelets; PT, prothrombin time; PTT, partial prothrombin time; CRP, C-reactive protein;

p values from paired Wilcoxon signed rank tests, corrected for multiple testing by multiplying the original p value by four, the number of time points, according to the Bonferroni method.

**Supplementary Figure 1:** Fibrinogen course at baseline (T1), on arrival at the ICU (T2), after 24 hours at the ICU (T3) and immediately before discharge from the ICU (T4) in the full unmatched population.

*: P<0.05; **: P<0.01; ***: P<0.001, NS, not significant (P values from Mann–Whitney U tests, corrected for multiple testing by multiplying the original p value by four, the number of time points, according to the Bonferroni method); points represent the median, bars the interquartile range.
