# Supplementary material for: Administration of fibrinogen concentrate combined with prothrombin complex maintains hemostasis in children undergoing congenital heart repair (a long‐term propensity score‐matched study)
Source: Acta Anaesthesiol Scand. 2021 Jul 26;65(9):1178–86. doi: 10.1111/aas.13945 (PMC8518112; doi:10.1111/aas.13945)
Supplement: Supplementary file 2 — Supplementary Table Oxygenator [file AAS-65-1178-s001.docx]

**Supplementary Table oxygenator:** Oxygenator types used during the study period

| Flow (L/min) | Year | Type of oxygenator | | |
| --- | --- | --- | --- | --- |
| 1.5 | 2000 – 2003  2003 - 2010  2010 - 2015 | DIDECO 901  CAPIOX RX05  CAPIOX FX05 |  | (Sorin Group S.R.L., Modena, Italy)  (Terumo Corporation, Tokyo, Japan)  (Terumo Corporation, Tokyo, Japan)  (Terumo Corporation, Tokyo, Japan)  (Terumo Corporation, Tokyo, Japan) |
| 1.5-2.8 | 2000 – 2006  2006 – 2015 | DIDECO 902  HILITE 2800 |  | (Sorin Group S.R.L., Modena, Italy)  (MEDOS Medizintechnik AG, Stolber, Germany) |
| 2.8-3.5 | 2000 – 2015 | DIDECO 705 |  | (Sorin Group S.R.L., Modena, Italy) |
| 3.0-6.0 | 2000 – 2001  2001 - 2015  2008 - 2010  2010 - 2015 | MONOLYTH  CAPIOX RX25  CAPIOX RX15  CAPIOX FX15 |  | (Sorin Group S.R.L., Modena, Italy)  (Terumo Corporation, Tokyo, Japan)  (Terumo Corporation, Tokyo, Japan)  (Terumo Corporation, Tokyo, Japan)  (Terumo Corporation, Tokyo, Japan)  (Terumo Corporation, Tokyo, Japan)  (Terumo Corporation, Tokyo, Japan) |
